# TRAF3/p38-JNK Signalling Crosstalk with Intracellular-TRAIL/Caspase-10-Induced Apoptosis Accelerates ROS-Driven Cancer Cell-Specific Death by CD40

**DOI:** 10.3390/cells11203274

**Published:** 2022-10-18

**Authors:** Khalidah Ibraheem, Albashir M. A. Yhmed, Mohamed M. Nasef, Nikolaos T. Georgopoulos

**Affiliations:** 1 Department of Biological Sciences, School of Applied Sciences, University of Huddersfield, Queensgate, Huddersfield HD1 3DH, UK; 2 Department of Medical Laboratory Sciences, Faculty of Medical Technology, Wadi Alshatti University, Wadi Alshatti P.O. Box 68, Libya

**Keywords:** CD40, apoptosis, ROS, carcinoma, TNFR, receptor crosstalk, TRAIL

## Abstract

The capacity to induce tumour-cell specific apoptosis represents the most unique feature of the TNF receptor (TNFR) family member CD40. Recent studies on the signalling events triggered by its membrane-presented ligand CD40L (mCD40L) in normal and malignant epithelial cells have started to unravel an exquisite context and cell type specificity for the functional effects of CD40. Here, we demonstrate that, in comparison to other carcinomas, mCD40L triggered strikingly more rapid apoptosis in colorectal carcinoma (CRC) cells, underpinned by its ability to entrain two concurrently operating signalling axes. CD40 ligation initially activates TNFR-associated factor 3 (TRAF3) and subsequently NADPH oxidase (NOX)/Apoptosis signal-regulating kinase 1 (ASK1)-signalling and induction of reactive oxygen species (ROS) to mediate p38/JNK- and ROS-dependent cell death. At that point, p38/JNK signalling directly activates the mitochondrial pathway, and triggers rapid induction of intracellular TNF-related apoptosis-inducing ligand (TRAIL) that signals from internal compartments to initiate extrinsic caspase-10-asscociated apoptosis, leading to truncated Bid (tBid)-activated mitochondrial signalling. p38 and JNK are essential both for direct mitochondrial apoptosis induction and the TRAIL/caspase-10/tBid pathway, but their involvement follows functional hierarchy and temporally controlled interplay, as p38 function is required for JNK phosphorylation. By engaging both intrinsic and extrinsic pathways to activate apoptosis via two signals simultaneously, CD40 can accelerate CRC cell death. Our findings further unravel the multi-faceted properties of the CD40/mCD40L dyad, highlighted by the novel TNFR crosstalk that accelerates tumour cell-specific death, and may have implications for the use of CD40 as a therapeutic target.

## 1. Introduction

The activation of CD40, a member of the TNF receptor (TNFR) superfamily by its cognate ligand CD154 (CD40L) is critical in the functioning of the immune system [1], as it is crucial in the stimulation of B-lymphocytes and antigen presenting cells, as well as in T-cell responses [2]. Moreover, CD40 is expressed by a variety of epithelial cell types [3], and accumulating evidence from investigations on the consequences of cell signalling triggered by the CD40L/CD40 dyad in epithelial cells has demonstrated that CD40 signalling can strongly influence non-lymphoid cell fate [4]. 

The ability of certain TNFRs (‘death receptor’ subfamily) to trigger cell death independently of protein synthesis induction is the TNFR family’s first cellular effect to be characterised in detail [5]. Several TNFRs (e.g., Fas, TRAIL-R1/DR4 and TRAIL-R2/DR5) can trigger rapid cell death, whereas others cause death less effectively (e.g., TWEAK, CD30) [6]. By contrast, certain TNFRs can act as ‘non-canonical’ apoptosis inducers by mechanisms that not only involve induction of protein synthesis but are also heavily dependent on cellular context (normal versus malignant cells) and the extent of receptor cross-linking (signal ‘quality’). CD40 epitomises such TNFRs [7,8] and until recently these properties and their underpinning mechanisms remained poorly defined, but recent investigations on CD40 have provided intriguing insights into the idiosyncratic, if not paradoxical [9], nature of non-canonical TNFR death signalling [4]. 

The exquisitely contextual, bimodal effect of CD40 ligation is evident not only in normal and malignant B-lymphocytes but also in epithelial cells; CD40 signalling is non-apoptotic (even cyto-protective) in normal cells, yet highly pro-apoptotic in their transformed counterparts [10,11,12]. Equally importantly, signal quality determines whether signalling is pro-apoptotic; receptor cross-linking by membrane-presented CD40L (mCD40L) causes extensive apoptosis, whilst soluble agonists cause little apoptosis and are mainly growth-inhibitory [13,14,15,16]. The importance of signal quality in functional outcome has also been highlighted in immune cells, where CD40 cross-linking defines B cell responses [17,18], whilst in dendritic cells both maturation and co-stimulatory activity [19], as well as the ability to induce direct anti-tumour effects [20], are associated with mCD40L (not soluble ligand). 

In carcinoma cells, CD40 recruits TNFR-associated factor (TRAF) proteins (such as TRAF3 [13]) to activate pro-apoptotic ASK-1 and facilitate activation of the NOX complex to induce ROS production [11]. This is followed by phosphorylation of JNK and p38 [12] and transcriptional regulation of pro-apoptotic mitochondrial proteins (Bak and Bax). In addition to ROS induction, mCD40L suppresses antioxidant mechanisms operating specifically in malignant epithelial cells, which pushes such cells past a “lethal pro-apoptotic threshold” and triggers carcinoma cell-specific death [11]. 

‘Classical’ death ligands (FasL and TRAIL) utilise canonical, pro-apoptotic pathways that rapidly recruit well-characterised signalling mediators [21] and may also link with mitochondrial apoptosis via Bid activation [22]. Yet, upon extensive cross-linking these receptors show significant toxicity to normal cells [23]. By contrast, mCD40L causes extensive apoptosis and despite cell death being as extensive as that triggered by Fas or TRAIL-R [10,13], apoptosis remains tumour cell-specific, yet it appears to progress less rapidly. Unlike death ligands which can trigger cell death within <24 h, we have previously shown that mCD40L-mediated killing in some epithelial types such as bladder and renal carcinoma cells is more prominently detected >24 h post-receptor ligation [10,14], with some pro-apoptotic markers (e.g., DNA fragmentation) showing maximal activation at 36–48 h [11,12]. 

However, one striking observation is that in colorectal carcinoma cells mCD40L-mediated death occurs more rapidly in comparison to other carcinoma cells, leading us to hypothesise that in these cells CD40 ligation may engage a more complex pathway. Here, we show that in these cells CD40 activates a dual signalling pathway that not only triggers TRAF3-p38/JNK driven induction of ROS which results in cytochrome c/caspase-9-associated apoptosis, but simultaneously rapidly upregulates intracellular TRAIL which causes Bid cleavage and caspase-10 (but not caspase-8)-dependent apoptosis. By inducing a divergent signalling pathway that partially utilises p38/JNK-induced TRAIL-dependent apoptosis, this crosstalk appears to amplify and accelerate CD40-mediated cell death. Our findings have unravelled more multifaceted properties for the CD40/CD40L dyad by providing novel evidence of direct TNFR crosstalk that drives rapid carcinoma cell-specific death. 

## 2. Materials and Methods

### 2.1. Cell Culture

The HCT116 cell line was obtained from the ATCC, supplied via Sigma (Sigma, Dorset, UK), and was maintained in DR medium (1:1 *v*/*v* mixture of DMEM and RPMI) supplemented with 5% FBS (Sigma). SW480-CD40 cells were established as previously [14] and were routinely maintained in DR/5% FBS medium supplemented with 1 mg/mL G418 (Invivogen, supplied by Source BioScience LifeSciences, Nottingham, UK) to ensure expression of the CD40 transgene. 3T3Neo and 3T3CD40L fibroblasts were established as detailed elsewhere [10] and maintained in DR/10% FBS and DR/10% FBS supplemented with 0.5 mg/mL G418, respectively, with omission of the antibiotic during co-culture experiments (below). 

### 2.2. CD40 Ligation

CD40 activation by mCD40L was carried out as detailed elsewhere [24]. Briefly, 3T3neo (control) and mCD40L-expressing 3T3CD40L (mCD40L) fibroblasts were growth-arrested by treatment with 10 µg/mL of Mitomycin C (MMC; Insight Biotechnology, Middlesex, UK) for 2 h in DR/5% medium and seeded either in 96-well plates at 1 × 10^4^ cells/well for apoptosis detection assays or 10 cm^2^ culture dishes at 3 × 10^6^ cells/dish for protein lysate preparation. Following overnight incubation, the culture medium was removed, and epithelial cells (HCT116 or SW480-CD40) were added at 1 × 10^4^ cells/well in 96-well plates or 3 × 10^6^ in 10 cm^2^ dishes, respectively. For some experiments, HCT116 cells had been transiently transfected or stably transduced to express siRNA and shRNA, respectively, as detailed below. 

### 2.3. Transient Transfection with siRNA

To knockdown TRAIL expression, HCT116 cells were transfected using the validated ON-TARGETplus SMARTpool™ Human TNFSF10 (8743) siRNA, Cat. No. L-011524-00-0005 (Dharmacon, supplied by Thermo Fisher Scientific, Loughborough, UK) or with the manufacturer’s recommended control siRNA. Cells were cultured in 25 cm^2^ flasks and transfected using DharmaFECT 2 Reagent in serum free medium (Thermo Fisher Scientific) according to the manufacturer’s instructions using siRNA at the final concentration of 50 nM. At 48 h post-transfection, cells were trypsinised and seeded appropriately for CD40 ligation (as above). 

### 2.4. Stable Expression of shRNA by Retroviral Transduction

For stable knockdown of Bid, the pSIREN-RetroQ vector system was used to generate anti-Bid shRNA retrovirus as described [11]. Two independent shRNA constructs were designed and the vector that showed optimal knockdown of Bid expression was the one targeting the previously verified sequence: 5′-GAAGACATCATCCGGAATA-3’ as confirmed by two independent publications [25,26]. Plasmid vector cloning, generation of recombinant retrovirus-expressing PT67 cells, collection of virus and transduction of HCT116 cells, and selection of stable anti-Bid and Control shRNA expressers in puromycin (Invivogen, supplied by Source BioScience LifeSciences, Nottingham, UK) was carried out as detailed elsewhere [11]. 

### 2.5. Cell Viability Assays 

HCT116 cells were seeded at 1 × 10^4^ cells/well in 96-well plates and treated with 10-20 ng/mL of human recombinant *killer*TRAIL™ (Enzo, Exeter, UK) in the absence or presence of anti-FasL mAb NOK-1, anti-TRAIL mAb RIK-2 or control isotype Control IgG (all at 10 µg/mL) [13], alongside untreated control cultures, for 24 h. Cell viability was then determined using the CellTiter 96^®^ AQueous One cell proliferation assay (Promega, Southampton, UK) by addition of 20 μL CellTiter reagent to each well and incubation at 37 °C for 4 h. Absorbance was measured using a FLUOstar OPTIMA (BMG Labtech, Bucks, UK) at 492 nm and percentage (%) Cell Viability calculated as previously described [27]. 

### 2.6. Detection of Cell Death 

In agreement with recommended guidelines regarding the use and interpretation of assays for monitoring cell death [28], a minimum of two assays were utilised for cell death detection: (a) CytoTox-Glo assay (Promega, Southampton, UK) for the detection of the compromising of cell membrane integrity, (b) SensoLyte Homogenous AFC Capase-3/7 assay (Anaspec, Cambridge Bioscience, Cambs, UK) for effector caspase-3/7 activation, and (c) DNA Fragmentation ELISA (Roche Diagnostics, West Sussex, UK) for detection of DNA fragment release. For DNA fragmentation, treatment with 5 µM staurosporine (Sigma) served as positive control and permitted calculation of % DNA fragmentation. The optimisation of these assays in the co-culture system of effector/3T3 and target/epithelial cells has been detailed elsewhere [24]. In some experiments, cell death detection in the presence of blocking antibodies NOK-1, RIK-2 and Control IgG (as detailed above) was carried out. 

### 2.7. Western Blotting 

Whole cell lysates were prepared from epithelial cells cultured alone or from co-cultures with 3T3Neo (controls) and 3T3CD40L (mCD40L-treated) cells and were denoted as ‘C’ and ‘mL’, respectively, in all figures. Lysate from effector (3T3CD40L) cells alone served as negative control (denoted as ‘NC’). Using lysates (20 µg and 40 µg protein/lane for monocultures and co-cultures, respectively) 4–12% SDS-PAGE and electroblotting were performed as detailed elsewhere [24]. For epithelial cell-alone lysates, antibodies used were: CD40 (sc-13128) (Insight Biotechnology) and β-actin (A5441) (Sigma). For co-culture lysates, antibodies used were: TRAF1 (sc-7186), TRAF3 (sc-949) and TRAF6 (sc-8409) (Insight Biotechnology); phospho-MKK4 (#4514), phospho-ASK1 (#3765), phospho-JNK (#9255), phospho-p38 (#9255), Thioredoxin-1 (#2285S), phospho-p40phox (#4311), DR5 (8074), TRAIL (#3219), FasL (#4273) and Bid (2002) (Cell Signalling Technologies, supplied by New England Biolabs, Herts, UK); Bak (AF816) and Bax (2282-MC-100) (R&D Systems). Antibody binding was detected using goat anti-rabbit IRDye^®^ 800 (Tebu-bio, Cambs, UK) or goat anti-mouse Alexa Fluor 680 (Thermo Fisher Scientific) secondary antibodies. For lysates prepared from 3T3neo/3T3CD40L (effector) and epithelial (target) cell co-cultures, sample loading was corrected/adjusted for epithelial cells according to reactivity with human-specific anti-cytokeratin 18 (CK18) antibody (C8541) (Sigma) and not using antibodies detecting non-phosphorylated intracellular proteins, as explained elsewhere [12,24]. For some experiments, mitochondrial and cytoplasmic fractions were isolated from co-cultures using the mitochondrial isolation kit (Millipore #MT1000) utilising primary antibodies supplied with the manufacturer’s kit (Millipore, Watford, UK), followed by appropriate secondary antibodies as detailed above. Immunolabelling was visualised using an Odyssey Infra-Red imaging system (LiCor, Cambs, UK). 

### 2.8. Functional Experiments Using Pharmacological Inhibitors

Inhibitors for AP-1 (NDGA), NADPH oxidase (NOX) (DPI) and the antioxidant N-acetyl L-cysteine (NAC) were from Sigma. JNK (SP600125), p38 (SB202190) and MEK/ERK (U0126) inhibitors were from Enzo. Inhibitors for caspase-8 (z-IETD-FMK), -9 (z-LEHD-FMK), -10 (z-AEVD-FMK) and pan-caspase inhibitor (z-VAD-FMK) were from R&D Systems (supplied by Bio-Techne, Abingdon, UK). NAC was reconstituted in DR/5% medium and its pH adjusted to 7.0 before use. All other inhibitors were reconstituted in DMSO (Sigma). Cells treated with DMSO alone (vehicle controls) were included in all experiments. 

### 2.9. ROS Detection

For detection of intracellular ROS, cells were treated with 1 M H2DCFDA (Thermo Fisher Scientific) for 30 min, fluorescence measurements (relative fluorescence units, RFU) were taken spectrophotometrically, and results expressed as fold increase in RFU as detailed previously [24]. For such experiments, effector (3T3Neo/3T3CD40L) cells were not growth-arrested (using MMC) to minimise background 3T3 cell-associated fluorescence. 

### 2.10. Flow Cytometry

CD40 expression by HCT116 and SW480-CD40 cells was detected using PE-conjugated mouse anti-human CD40 antibody and an isotype control IgG1-PE (BD Biosciences, Berks, UK). In all, 10,000 events were acquired on a Guava EasyCyte flow cytometer and results analysed using EasyCyte software (Luminex, Amsterdam, The Netherlands).

### 2.11. Statistics

Mean values and standard error of the mean (SEM) were used as descriptive statistics. Two-tailed, paired or non-paired Student’s *t*-tests were used for evaluation of statistical significance. For graphical purposes in the figures, significance symbols used are as follows: * *p* < 0.05, ** *p* < 0.01 and *** *p* < 0.001, whilst NS denotes non-significance (*p* > 0.05).

## 3. Results

### 3.1. Ligation of CD40 by mCD40L Induces Rapid Apoptosis in Colorectal Cancer (CRC) Cells in Comparison to Other Carcinoma Cell Types

**Figure 1:**
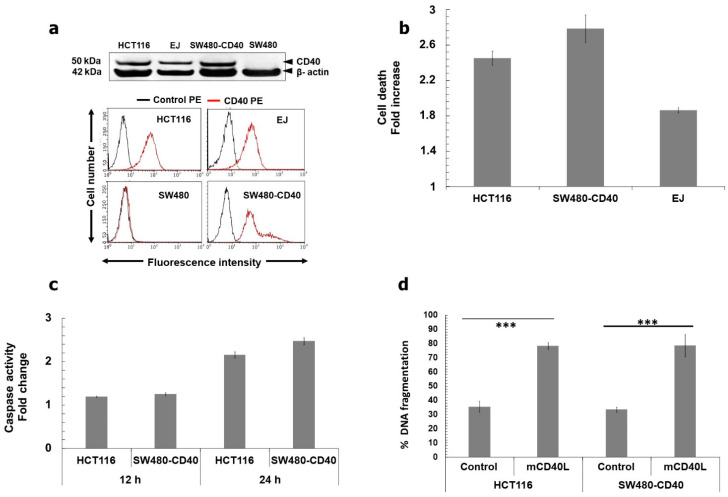
Membrane-CD40L (mCD40L) induces rapid apoptotic death in CRC cells. (**a**) Immunoblotting images (upper panels) show detection of CD40 expression in CRC lines HCT116, SW480 and SW480-CD40. Correct cell lysate loading was confirmed by β-actin detection. Flow cytometry (lower panels) was used to determine surface expression of CD40 by CRC lines. Overlay histogram plots show CD40-PE (red histograms) versus control PE-conjugated (black histograms) antibody labelling. EJ and SW480 cells served as CD40+ve and CD40−ve controls, respectively, for immunoblotting and flow cytometry experiments. (**b**) HCT116, SW480-CD40 and EJ cells were co-cultured with 3T3Neo (Control) or 3T3CD40L (mCD40L) effector cells for 24 h and death was detected using the CytoTox-Glo assay. Cell death was detected as background-corrected relative luminescence units (RLU) and results are presented as Cell death Fold increase in RLU readings (mCD40L relative to Control), and are representative of at least 3 independent experiments. Bars show mean fold increase in RLU for 5–6 technical replicates ± SEM. (**c**) CRC cells were treated with mCD40L for 12 and 24 h by co-culture performed as described in ‘b’ and effector caspase-3/7 activity was assessed using the SensoLyte caspase-3/7 assay. Caspase activity was detected as background-corrected relative fluorescence units (RFU), and results are presented as Caspase activity Fold increase in RFU readings (mCD40L relative to control) and are representative of 3 experiments. Bars show mean fold change in caspase activity for 5–6 technical replicates ± SEM. (**d**) HCT116 and SW480-CD40 cells were treated with mCD40L (as above) for 24 h alongside appropriate negative controls (and positive control, staurosporine-treated cells) and DNA fragmentation was assessed using a DNA Fragmentation ELISA. Results are presented as % DNA fragmentation relative to positive control (maximal DNA fragmentation) and are representative of 3 experiments. Bars show mean % DNA fragmentation for 5–6 technical replicates ± SEM. *** *p* < 0.001.

We have previously demonstrated that mCD40L causes extensive apoptosis in colorectal carcinoma (CRC) cells that are naturally CD40+ve (HCT116 cells), as well as in CD40−ve cells in which CD40 expression was restored by retroviral transduction (SW480-CD40 cells) [14]. Figure 1a confirmed CD40 expression in HCT116 and SW480-CD40 cells. Although mCD40L caused death that was quantitively comparable to other cell types (bladder/UCC and renal/RCC) at 36–48 h [11,12], we noticed that in CRC cells CD40-mediated apoptosis was efficiently being triggered much earlier. mCD40L caused plasma membrane integrity disruption at 24 h far more effectively in HCT116 and SW480-CD40 cells than in CD40+ve UCC cells EJ (Figure 1b) and RCC cells 786-O (not shown). In fact, cell death was detectable as early as 12 h post receptor ligation in HCT116 cells treated with mCD40L (not shown). Concordantly, we observed effector caspase-3/7 activation and DNA fragmentation (Figure 1c,d) triggered by mCD40L within 24 h. By contrast, no or little such activation was observed in EJ and 786-O cells (not shown), in agreement with studies where these apoptotic markers were more readily detectable at 48 h post mCD40L treatment [11,12]. Of note, mCD40L-mediated apoptosis was at least as extensive in SW480-CD40 cells as in HCT116 cells; therefore, despite loss of CD40 expression during carcinogenesis, SW480 cells maintained the apoptotic signalling effectors utilised by CD40 to trigger rapid cell death. Overall, these findings suggested that mCD40L engages a pro-apoptotic pathway that causes more rapid death in CRC cells in comparison to other carcinoma types. 

### 3.2. mCD40L-Mediated Regulation of TRAF Adaptor Protein Expression and Activation of Pro-Apoptotic MAP Kinases 

**Figure 2:**
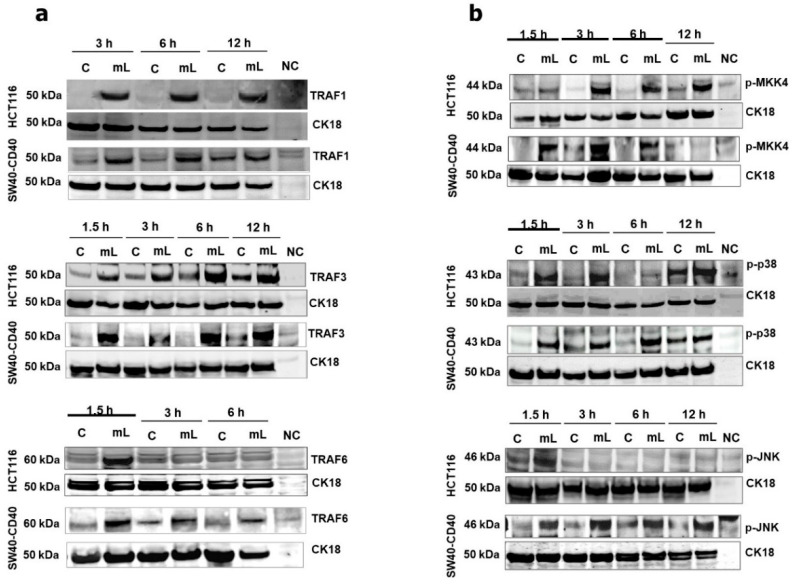
Regulation of TRAF adaptor proteins and pro-apoptotic MAP kinases following CD40 ligation by mCD40L in CRC cells. (**a**) TRAF1, TRAF3 and TRAF6 protein expression was detected by immunoblotting following co-culture of HCT116 and SW480-CD40 cells with 3T3-Neo [Control (C)] or 3T3-CD40L [mCD40L (mL)] at the indicated time points. Lysate from effector (3T3-CD40L) cell monocultures served as negative control (NC) to confirm the human-protein specificity of the antibodies. Equal loading for human epithelial cell lysate was confirmed by CK18 detection (as detailed in the Methods). (**b**) Phosphorylation of MKK4 (p-MKK4), p38 (p-p38) and JNK (p-JNK) was detected by immunoblotting following treatment of HCT116 and SW480-CD40 cells with mCD40L (mL) versus control (C) as in (**a**).

We have recently reported that mCD40L differentially regulates TRAF adaptor protein expression in normal and malignant epithelial cells, with TRAF3 stabilisation being central in the induction of apoptosis in carcinoma cells [11]. mCD40L rapidly induced the expression of TRAF1 and TRAF3 proteins in both HCT116 and SW480-CD40 cells (Figure 2a), whereas no clear changes in TRAF2 expression were detectable (not shown). TRAF1 and particularly TRAF3 induction was rapid and sustained throughout the course of mCD40L treatment, and TRAF3 was critical in apoptosis as shRNA-mediated TRAF3 knockdown attenuated cell death (Supplementary Figure S1). Notably, mCD40L also triggered TRAF6 upregulation, which was transient, as expression subsided completely in HCT116 cells within 3 h and was dramatically reduced in SW480-CD40 by 3-6h post CD40 ligation (Figure 2a). Furthermore, mCD40L triggered rapid and transient induction of phosphorylated MKK4 in both HCT116 and SW480-CD40 cells (Figure 2b). By contrast, mCD40L mediated the rapid and sustained phosphorylation of p38 whilst also inducing JNK phosphorylation, the latter being more prominent and sustained in SW480-CD40 cells (Figure 2b). 

### 3.3. mCD40L Activates NADPH Oxidase (NOX) and ASK1 to Trigger a NOX-Mediated, ROS-Dependent Apoptotic Pathway

**Figure 3:**
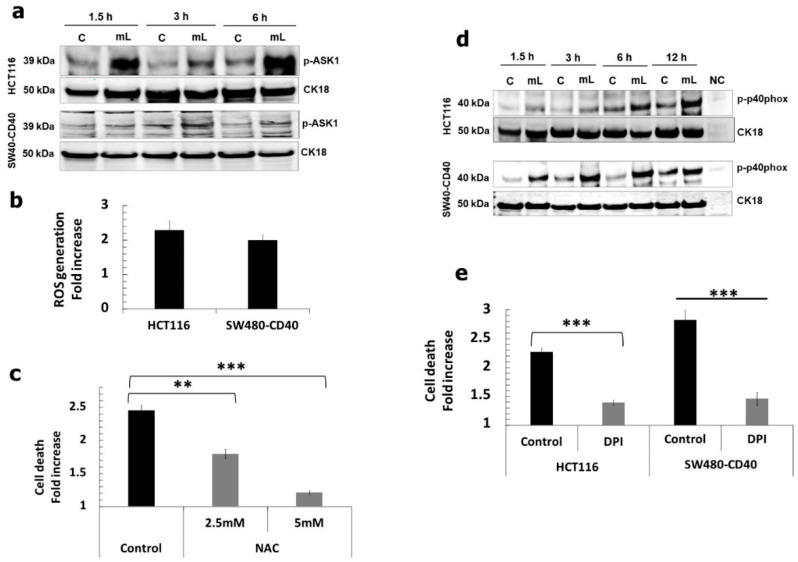
Induction of ROS- and NADPH oxidase-associated signalling pathways and their functional role in mCD40L-mediated CRC cell apoptosis. (**a**) Expression of phosphorylated ASK1 (p-ASK1) was detected in controls (‘C’) versus mCD40L-treated (‘mL’) HCT116 and SW480-CD40 cells by immunoblotting at the indicated time points. Equal loading for human epithelial cell lysate was confirmed by CK18 detection. (**b**) HCT116 and SW480-CD40 cells were treated with or without mCD40L for 0.5 h and intracellular ROS was detected using 0.5 µM H2DCFDA. Background-corrected relative fluorescence units (RFU) obtained were used to present the data as ROS generation fold increase in RFU readings (mCD40L relative to control). Results are representative of 2 experiments. Bars show mean fold increase for 5–6 technical replicates ± SEM. (**c**) HCT116 cells were treated with mCD40L in the absence (vehicle control–denoted ‘Control’) or in the presence of the indicated concentration of ROS inhibitor NAC. Cell death was detected at 24 h using the CytoTox-Glo assay. Results are presented as Cell death fold increase in background-corrected RLU relative to control (mCD40L treatment versus controls) and are representative of at least 2 independent experiments. Bars show mean fold change (comparing NAC-treated versus vehicle control cultures) for 4–5 technical replicates ± SEM. (**d**) Expression of phosphorylated p40phox (p-p40phox) was detected in controls (‘C’) versus mCD40L-treated (‘mL’) HCT116 and SW480-CD40 cells by immunoblotting. Lysate from effector (3T3CD40L) cell monocultures served as negative control (NC). Equal loading for human epithelial cell lysate was confirmed by CK18 detection. (**e**) HCT116 cells and SW480-CD40 cells were treated with mCD40L in the absence (vehicle control–denoted ‘Control’) or presence of 0.125 µM of NOX inhibitor DPI. Cell death was detected at 24 h using the CytoTox-Glo assay. Results are presented as Cell death fold increase in background-corrected RLU relative to control (mCD40L treatment versus controls) and are representative of 3 independent experiments. Bars show mean fold change (comparing DPI-treated versus vehicle control cultures) for 4-5 technical replicates ± SEM. ** *p* < 0.01 and *** *p* < 0.001.

We then sought to delineate CD40 receptor-proximal signalling mediators that directly link the TRAF proteins with downstream p38/JNK-related cellular responses. We found that mCD40L triggered phosphorylation of the apoptosis-signal regulating kinase 1 (ASK1) which remained phosphorylated even after 6 h post-receptor ligation, particularly in HCT116 cells (Figure 3a); interestingly, unlike our previous observation in bladder and renal cancer cells, the activation of ASK1 was more rapid and exhibited a bi-phasic pattern (evident at 1.5 and 6 h). ASK1 acts as a ‘master redox sensor’ during apoptosis; its activation coincides with induction of ROS, oxidation of its inhibitor Thioredoxin (Trx) and its subsequent physical dissociation from Trx. Indeed, we found that Trx-1 protein levels progressively increase in SW480-CD40 and HCT116 cells during culture, but CD40 activation attenuated Trx-1 expression particularly in HCT116 cells (Supplementary Figure S2). In parallel, we found that mCD40L caused the induction of intracellular ROS within 1h (Figure 3b) which was essential in CD40-mediated death, as ROS blockade using N-acetyl-cysteine (NAC) dose-dependently abrogated apoptosis (Figure 3c). 

As the NADPH oxidase (NOX) cell membrane complex represents one major source for rapid, receptor-mediated induction of intracellular ROS release and NOX activation has been implicated in CD40-mediated apoptosis [11,12], we hypothesised that NOX may be activated and functionally involved in apoptosis. mCD40L caused rapid phosphorylation of the NOX-2 regulatory subunit p40phox and this was sustained even after 12 h post receptor ligation (Figure 3d) which was much more rapid that the p40phox activation in other types of carcinoma cells [12]. Having shown that apoptosis is ROS dependent (Figure 3c), we also found that the NOX inhibitor diphenyleneiodonium (DPI) blocked mCD40L killing to the same extent as NAC treatment (Figure 3e). Strikingly also, NOX inhibition by DPI interfered with mCD40L-mediated signalling by attenuating TRAF3 induction and downstream pro-apoptotic MAPK signalling (Supplementary Figure S3). Collectively, these findings suggest that NOX-generated ROS is critical in mCD40L-mediated signalling and apoptosis. 

### 3.4. mCD40L-Mediated Apoptosis Is Amplified and Accelerated by Partial Crosstalk with Intracellular TRAIL-/Caspase-10-Mediated Bid Activation

Although the early signalling events triggered mCD40L in CRC cells were similar in nature to those we observed in other carcinoma cell types, the timing of these events (particularly ASK1 activation and NOX-mediated ROS induction) was more rapid. Yet, we hypothesised that simply the speed of the receptor-proximal signalling events may not mechanistically explain the strikingly more potent and rapid pro-apoptotic mCD40L signal in CRC cells. 

**Figure 4:**
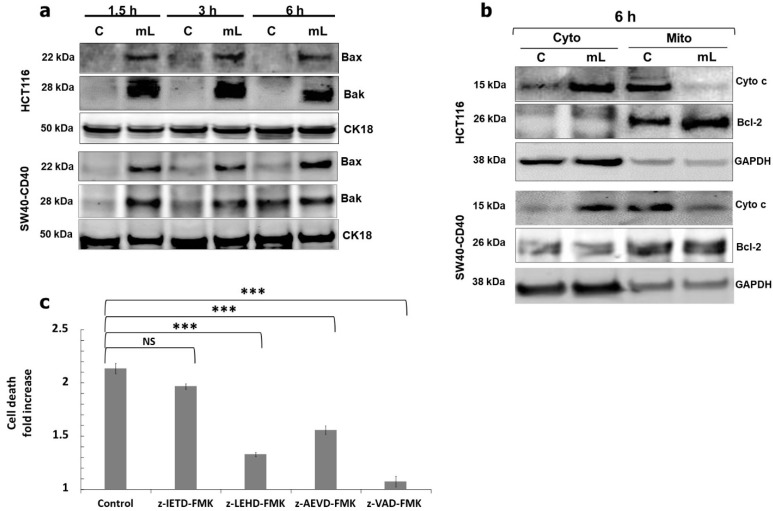
Rapid induction of the mitochondrial (intrinsic) apoptotic pathway and role of caspase activation in cell death. (**a**) Expression of Bax and Bak proteins was detected in controls (‘C’) versus mCD40L-treated (‘mL’) HCT116 and SW480-CD40 cells by immunoblotting at the indicated time points. Equal loading for human epithelial cell lysate was confirmed by CK18 detection. (**b**) Control (‘C’) and mCD40L-treated (‘mL’) HCT116 and SW480-CD40 cells were used to prepare cytoplasmic (‘Cyto’) and mitochondrial (‘Mito’) sub-cellular fractions for the detection of cytochrome c (Cyto c) protein by immunoblotting at 6 h post CD40 ligation. Detection of Bcl-2 and GAPDH proteins was employed to confirm sub-cellular fractionation (mitochondrial and cytoplasmic, respectively). (**c**) HCT116 cells were treated with mCD40L in the absence (vehicle control–denoted ‘Control’) or presence of 100 µM inhibitor of caspase-8 (z-IETD-FMK), caspase-9 (z-LEHD-FMK), caspase-10 (z-AEVD-FMK) or pan-caspase inhibitor (z-VAD-FMK). Cell death was detected 24 h later using the CytoTox-Glo assay. Results are presented as Cell death Fold increase in background-corrected RLU readings relative to control (mCD40L treatment versus controls) and are representative of 3 independent experiments. Bars show mean fold change (comparing caspase inhibitor-treated versus vehicle control cultures) for 5–6 technical replicates ± SEM. NS denotes non-significance (*p* > 0.05) and *** *p* < 0.001.

We thus investigated more distal downstream mediators of the CD40 signalling pathway. Being a non-classical TNFR, CD40 has been reported to engage the intrinsic, mitochondrial pathway of apoptosis which requires >6–12 h before Bak/Bax induction and mitochondrial outer membrane permeabilization (MOMP) as observed in some cancer cell types [11,12]. Strikingly, however, mCD40L induced rapid Bak and Bax induction in CRC cells by 1.5h post CD40 ligation which appeared maximal (Figure 4a). Consequently, the mitochondrial pathway was fully activated within <6 h post mCD40L treatment, evident by the cytoplasmic release of Cytochrome c and the near-complete loss of mitochondrial Cytochrome c (Figure 4b). Bax induction was critical in apoptosis, as shRNA-mediated Bax knockdown abrogated mCD40L-induced cell death (Supplementary Figure S4). 

As this strikingly rapid nature of mCD40L killing in CRC cells was highly reminiscent of the speed of apoptosis triggered by some ‘classical’ death TNFR ligands, we examined whether mCD40L-mediated apoptosis may involve (at least in part) the extrinsic apoptotic pathway. Using pharmacological intervention, we observed that both pan-caspase inhibition (using zVAD) and inhibition of caspase-9 caused nearly complete blockade of mCD40L-mediated apoptosis. Additionally, although caspase-8 inhibition had little effect on CD40-killing, unexpectedly, blockade of caspase-10 attenuated cell death by ~50% (Figure 4c). Thus, the rapidity of mitochondrial MOMP induction for Cyto c release and caspase-9 activation, together with the observation that caspase-10 is functionally important in apoptosis, implied mCD40L signalling crosstalk with the extrinsic, death receptor pathway. 

**Figure 5:**
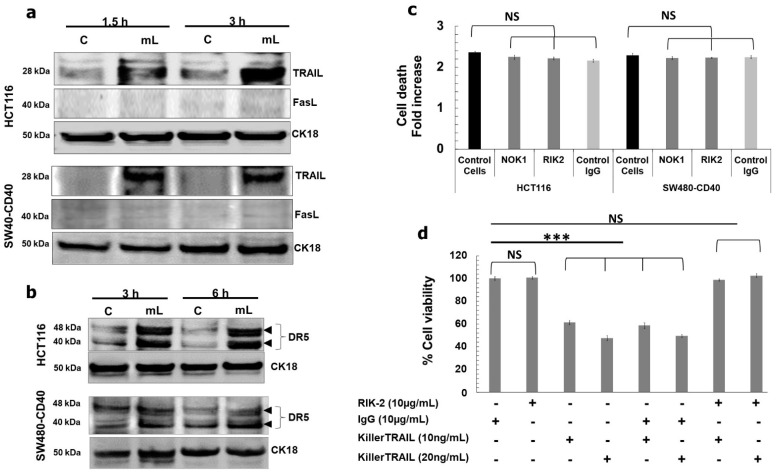
mCD40L-mediated signalling induces rapid up-regulation of DR5 receptor and induction of intracellular TRAIL expression. (**a**) Expression of TRAIL and FasL proteins was detected in controls (‘C’) versus mCD40L-treated (‘mL’) HCT116 and SW480-CD40 cells by immunoblotting at the indicated time points. Equal loading for human epithelial cell lysate was confirmed by CK18 detection. (**b**) DR5 receptor expression in HCT116 and SW480-CD40 cells at the indicated time points post CD40 ligation was detected by immunoblotting as in (**a**). (**c**) HCT116 and SW480-CD40 cells were treated with or without mCD40L in the presence of 10 μg/mL anti-FasL mAb (NOK1), anti-TRAIL mAb (RIK2) or isotype control mAb (Control Ig) and cell death was detected at 24 h using the CytoTox-Glo assay. Results are presented as Cell death fold increase in background-corrected RLU relative to control (mCD40L treatment versus controls) and are representative of 2 independent experiments. Bars show mean fold change (comparing mAb-treated cells versus untreated ‘Control cells’) for 4–5 technical replicates ± SEM. NS denotes non-significance (*p* > 0.05). (**d**) HCT116 cells were treated with the indicated concentration of killerTRAIL for 24 h in the presence of anti-TRAIL mAb (RIK-2) or isotype control mAb (Control Ig) and cell viability was assessed using the CellTiter 96 AQueous One Solution Cell Proliferation Assay. Results are presented as % viability for all experimental conditions relative to untreated (control) cells. Bars show mean % Cell Viability for 5–6 technical replicates ± SEM. NS denotes non-significance (*p* > 0.05) and *** *p* < 0.001.

**Figure 6:**
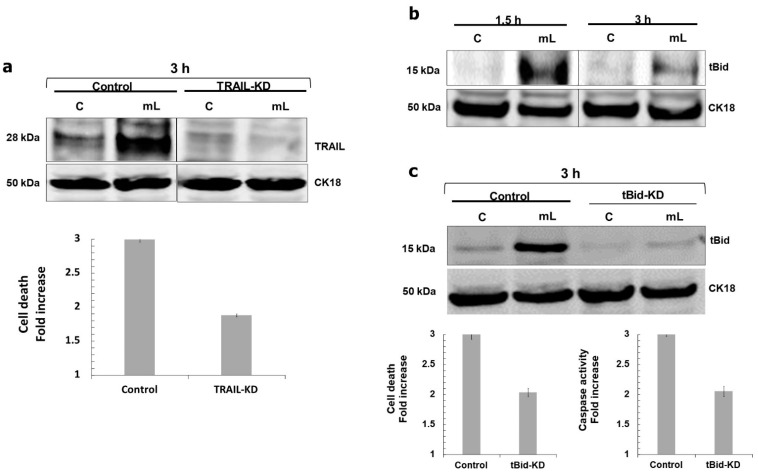
mCD40L induces Bid cleavage and knockdown of TRAIL or Bid partially attenuates mCD40L-mediated apoptosis. (**a**) HCT116 cells were transiently transfected for 24 h with anti-TRAIL siRNA (TRAIL-KD) or scrambled control siRNA (Control), and TRAIL protein expression was detected in untreated (‘C’) versus mCD40L-treated (‘mL’) TRAIL-KD and Control cells at 3 h by immunoblotting. Equal loading for human epithelial cell lysate was confirmed by CK18 detection (upper panels). TRAIL-KD and Control siRNA HCT116 cells were treated with mCD40L (‘mL’) alongside untreated control cultures (‘C’) and cell death was detected at 24 h using the CytoTox-Glo assay. Results are presented as Cell death fold increase in background-corrected RLU relative to control (mCD40L versus controls) and are representative of 3 experiments. Bars show mean fold change for 5–6 technical replicates ± SEM (lower panel). (**b**) Expression of cleaved Bid (tBid) protein was detected in controls (‘C’) versus mCD40L-treated (‘mL’) HCT116 cells by immunoblotting at the indicated time points. Equal loading for human epithelial cell lysate was confirmed by CK18 detection. (**c**) HCT116 cell derivatives that stably express anti-tBid shRNA (tBid-KD) as well as isogenic controls (Control) cells were established by retrovirus transduction. Expression of tBid was detected in control (‘C’) versus mCD40L-treated (‘mL’) tBid-KD and isogenic Control cells at 3 h by immunoblotting. Equal loading for human epithelial cell lysate was confirmed by CK18 detection (upper panels). tBid-KD and isogenic Control HCT116 cells were treated with mCD40L (‘mL’) alongside control cultures (‘C’), and cell death (left lower panel) or effector caspase-3/7 activity (right lower panel) were assessed 24 h and 48 h later using CytoTox-Glo and SensoLyte caspase-3/7 assays, respectively. For death detection, results are presented as Cell death fold increase in background-corrected RLU relative to control (mCD40L treatment versus controls). Caspase activity was detected as background-corrected relative fluorescence units (RFU), and results are presented as Caspase activity Fold increase in RFU readings (mCD40L relative to control). Bars show mean fold change for 5–6 technical replicates ± SEM.

We found that mCD40L triggered upregulation of TRAIL (but not FasL) in both HCT116 and SW480-CD40 cells as early as 1.5 h post treatment (Figure 5a), which was accompanied by up-regulation of the TRAIL-R2 receptor DR5 (Figure 5b). Moreover, we observed induction of Bid cleavage to tBid at the same time (Figure 6b). To test whether mCD40L-induced TRAIL was involved in apoptosis, we used the well-characterised TRAIL-blocking mAb RIK-2 [13] during mCD40L treatment of CRC cells. Surprisingly, we found that RIK2 had no effect on cell death (Figure 5c), and neither did the antagonistic anti-FasL mAb NOK1 concordantly with the lack of any induction of FasL (Figure 5a). We confirmed the functionality of the RIK2 mAb by demonstrating that RIK2 could completely block the loss of cell viability induced by exogenous, recombinant killerTRAIL in HCT116 cells (Figure 5d); therefore, cell-surface expressed TRAIL could not be linked with the induction of Bid-associated mitochondrial apoptosis. Notably, however, we found that siRNA-mediated knockdown of TRAIL attenuated mCD40L-mediated apoptosis by ~50% (Figure 6a). Concordantly, TRAIL knockdown ablated Bid cleavage and shRNA-mediated tBid knockdown also caused a ~50% reduction in both apoptosis induction and caspase-3/7 activation (Figure 6c). Collectively, these findings, combined with the observation that pharmacological inhibition of caspase-10 attenuated cells death by ~50%, provided strong evidence that mCD40L-mediated apoptosis involves partial crosstalk with intracellular TRAIL-mediated activation of the mitochondrial apoptotic pathway, which amplifies and accelerates CD40-mediated death in CRC cells. 

### 3.5. Role of TRAF3 and Pro-Apoptotic p38 and JNK Kinases in mCD40L-Mediated Intrinsic and Extrinsic Cell Death Signalling 

**Figure 7:**
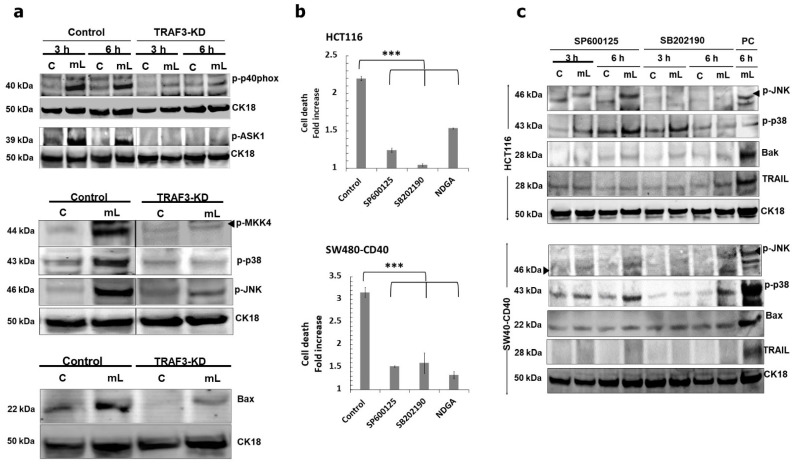
Role of TRAF3 and pro-apoptotic p38 and JNK kinases in mCD40L-mediated intrinsic and extrinsic cell death signalling pathways. (**a**) Retrovirus transduction was used to establish HCT116 cell derivatives that stably express anti-TRAF3 shRNA (TRAF3-KD) as well as isogenic controls (Control) cells. Expression of activated p40phox (p-p40phox), phosphorylated MAP kinases ASK1 (p-ASK1), MKK4 (p-MKK4), p38 (p-p38), JNK (p-JNK), and pro-apoptotic Bax was detected in untreated (‘C’) versus mCD40L-treated (‘mL’) TRAF3-KD and Control HCT116 cells at 3 h by immunoblotting (for p-p40phox and p-ASK1 detection experiments results for 6 h are also shown). Equal loading for human epithelial cell lysate was confirmed by CK18 detection in all experiments. (**b**) HCT116 and SW480-CD40 cells were treated with mCD40L in the absence (vehicle control–denoted ‘Control’) or presence of 5 µM of JNK inhibitor SP600125, p38 inhibitor SB202190, or AP-1 inhibitor NDGA. Cell death was detected at 24 h using the CytoTox-Glo assay. Results are presented as Cell death fold increase in background-corrected RLU relative to control (mCD40L treatment versus untreated controls) and are representative of 3 independent experiments. Bars show mean fold change (comparing inhibitor-treated versus vehicle control cultures) for 4–5 technical replicates ± SEM. *** *p* < 0.001. (**c**) Untreated (‘C’) and mCD40L-treated (‘mL’) HCT116 and SW480-CD40 cells in the presence of 5µM JNK inhibitor SP600125 or p38 inhibitor SB202190 were used to detect phosphorylated JNK (p-JNK) and p38 (p-p38), pro-apoptotic Bak or Bax, and TRAIL protein by immunoblotting at 3 h and 6 h post CD40 ligation. Lysates from HCT116 and SW480-CD40 cell cultures treated with mCD40L for 6 h in the absence of inhibitor were included (denoted as positive control, ‘PC’) for each experiment, respectively. Equal loading for human epithelial cell lysate was confirmed by CK18 detection in all experiments.

Following on from the original observation that CD40 ligation can induce intracellular TRAIL-mediated partial crosstalk with the mitochondrial apoptotic pathway, we aimed to establish a functional link between receptor proximal signalling events and activation of downstream mediators of apoptosis. Having demonstrated the critical role of TRAF3 in apoptosis above (Supplementary Figure S1), we found that TRAF3 knockdown fully abrogated induction of ASK1, p38 and JNK as well as subsequent induction of Bax, which occurred in parallel with the attenuation of p40phox phosphorylation (Figure 7a). Therefore, loss of TRAF3 results in the disruption of what would be a TRAF3/NOX signalling complex that can trigger activation of ASK1 and subsequently MKK4 and p38/JNK. Both p38 and JNK were functionally essential in the induction of apoptosis in both HCT116 and SW480-CD40 cells and so was the AP-1 transcription factor, as shown by functional blocking experiments using appropriate pharmacological inhibitors (Figure 7b). 

Our findings above showed that phosphorylation of p38 was more rapid as well as more sustained in comparison to JNK activation (Figure 2b). Yet, it remained unclear whether p38 and JNK operated independently, or there was functional (in addition to temporal) hierarchy in their activation. Using both HCT116 and SW480-CD40 cells, we found that although pharmacological blockade of JNK attenuated its phosphorylation, this had no effect on p38 phosphorylation; by contrast, specific pharmacological p38 inactivation not only suppressed p38 phosphorylation but it also fully abrogated induction of phospho-JNK (Figure 7c). Of note, functional inhibition of both p38 and JNK blocked the induction of TRAIL as well as suppressing induction of pro-apoptotic Bax/Bak. Therefore, although both p38 and JNK are key mediators of apoptosis as well as being crucial for the induction of TRAIL-associated crosstalk with mitochondria, p38 phosphorylation precedes JNK activation. 

## 4. Discussion

Cell surface-presented (membrane-bound) forms of TNF ligands (mTNFs) are superior in their capacity to induce receptor clustering in comparison to soluble forms. This is most likely due to positioning of mTNFs and their cognate receptors within membrane lipid rafts which allows formation of larger aggregates, stabilises ligand-receptor binding and dictates higher-order organisation/recruitment of signalling proteins [5]. The cellular functions activated by TNFRs differ in their dependence on such higher-order signalling-protein organization, with membrane-bound and soluble forms of TNF ligands having differential abilities to trigger cellular responses by causing different extents of receptor cross-linking [29]. This critical role of signal ‘quality’ in functional outcome is particularly evident in the case of ‘non-canonical’ pro-apoptotic TNFRs, as epitomised by the CD40 receptor. 

Classical death receptors activated by their ligands (e.g., FasL and TRAIL) utilise ‘canonical’, pro-apoptotic pathways that recruit well-characterised signalling mediators (as exemplified by the death inducing signalling complex) [21]; additionally, these receptors can link the extrinsic pathway with mitochondrial (intrinsic) apoptosis via Bid cleavage to amplify apoptosis [22]. Yet, upon extensive cross-linking or membrane-presentation [30], these death receptors show significant normal cell cytotoxicity [31], such as in hepatic [32] and bladder epithelial (urothelial) cells [33]. mCD40L represents the ‘natural’ ligand for its cognate receptor CD40, evident in the context of its immune cell function [34]; whereas cleaved, soluble CD40L appears to play different physiological roles as well as being detectable and/or involved mainly in pathological conditions [35,36]. Our previous studies have demonstrated that CD40L membrane-bound presentation transforms what is a weak signal in epithelial cells into a potent pro-apoptotic mediator that remains tumour cell-specific [11,12]. 

Whilst delineating the signalling events of mCD40L-mediated signalling, we observed induction and sustained upregulation of TRAF1, which is a marker of functional CD40 expression in epithelial cells [12,13]. Interestingly we also observed TRAF6 induction, however TRAF6 expression was transient and gradually diminished over time along the course of apoptosis, in agreement with previous observations reporting TRAF6 down-regulation during pro-apoptotic CD40 ligation [37]. Importantly, TRAF3 was rapidly induced and was essential in pro-apoptotic signalling, particularly the induction of p38 and JNK, thus: (a) supporting our previous observations on TRAF3’s prominent role at the heart of CD40 pro-apoptotic signalling [11], (b) providing further evidence for the emerging role of TRAF3 as a tumour suppressor from the work by Bishop and colleagues in normal and malignant B lymphocytes [38], and (c) in agreement with recent elegant studies by Xie and colleagues that have provided evidence for the versatility of TRAF3 in activating the mitochondrial apoptotic pathway [39]. 

We have recently shown that mCD40L stabilises the TNFR-associated factor (TRAF) protein TRAF3, which is critical in the activation of the redox sensor ASK-1. We also noted a biphasic ROS response and subsequent phosphorylation of pro-apoptotic p38 and JNK, the activation of which is temporally controlled and drives transcriptional regulation of MOMP-mediating mitochondrial proteins Bak and Bax [12]. Our current findings have provided evidence for rapid activation of the NOX complex and bi-phasic ASK1 activation which, in combination with our previous observations, strongly support the notion that mCD40L triggers a ‘two-step’ ROS response. This involves initial activation of the NOX complex to induce the first ‘wave’ of ROS production [12], followed by p38/JNK-mediated Bak/Bax activation and mitochondrial MOMP induction, and an anticipated secondary ROS release [11] leading to caspase-9 activation and apoptosis. 

Another directly related interesting notion raised by the present study relates to TRAF3 activation and the potential importance of ROS and/or the NOX complex in this process. Our previous work has implied that TRAF3 becomes rapidly stabilised following CD40 cross-linking by mCD40L [13], yet the precise mechanisms of this remain unclear. Here, we found that NOX activation in CRC cells is critical in TRAF3 induction, as pharmacological blockade of NOX attenuated stabilisation of TRAF3 and JNK phosphorylation, and ablated apoptosis evident by the loss of Bax induction (Supplemental Figure S3). Additionally, in light of the observation in other cancer cell types that it is NOX function, but not NOX-mediated ROS production per se, that facilitates TRAF3 induction [11], it is tempting to speculate that rapidly after CD40 cross-linking by mCD40L, direct association (recruitment) of NOX and its catalytic components (including p40phox) with CD40 leads to formation of a pro-apoptotic multi-protein complex. Such a ‘CD40 signalosome’ may then drive receptor-proximal signalling events, and work is currently underway in our laboratory to address these compelling hypotheses. 

One interesting question raised by our present studies concerns the high susceptibility of CRC cells to mCD40L-mediated death in comparison to other carcinoma cell types. In fact, the idiosyncratic, normal physiology of the intestinal and colonic epithelia may provide clues for this susceptibility. From a physiology point of view, the gut epithelium is a tissue where the ‘default’ cellular response is apoptosis, which underpins its particularly high turn-over rate, whilst TNF members can be key drivers in the perturbation of normal homeostasis resulting in pathology (mainly inflammatory disease) where excessive apoptosis is observed [40]. Concordantly, concerted dysregulation of several pro-apoptotic mediators is strongly linked with the development of CRC, which permits the suppression of cell death in malignant colonocytes [41]; with loss of CD40 expression in CRC being regarded as an important contributing factor towards apoptosis- and immune-evasion [14]. Collectively, these observations suggest that the gut epithelium naturally exhibits particularly high susceptibility to cell death signals. With its ability to trigger a dual ‘hit’ (with cross-talking extrinsic and intrinsic apoptotic pathways), mCD40L appears to exploit this susceptibility and induce rapid cell death, whilst apoptosis is equally efficient following restoration of CD40 expression (SW480-CD40 cells). Of equally relevant note, more intriguing clues for such a tissue-specific susceptibility to CD40 are apparent from the pioneering work by Afford and colleagues and their previous studies on the mechanisms of CD40-mediated control of liver homeostasis [32]; their work demonstrated that CD40 ligation may lead to hepatocyte toxicity via induction of extracellular (NOK1-inhibitable) FasL-mediated apoptosis [42]. Therefore, although in most epithelial cell types CD40 triggers a direct, non-canonical pro-apoptotic pathway, the observations for specific crosstalk in liver and colorectal cells, adds to the remarkable context- and cell type-specificity of the CD40/CD40L dyad. 

Contrary to other carcinoma types, mCD40L triggered noticeably more rapid apoptosis in CRC cells. Although the early signalling events (TRAF adaptor protein upregulation, NOX-mediated ROS release and downstream activation of pro-apoptotic MAPKs) were similar in nature to those we observed in other cell types, the timing of these events (exemplified by the activation of ASK1) in CRC cells was faster. To mechanistically explain the more rapid pro-apoptotic mCD40L signal in CRC cells, we investigated more distal signalling events. For the first time, we show that in order to induce Bak/Bax and subsequently trigger MOMP, CD40-mediated signalling entrains TRAIL-induced apoptosis. Interestingly, we observed rapid induction of TRAIL which was accompanied by an increase in expression of TRAIL-R2 (but no increase in TRAIL-R1 expression—not shown). However, no TRAIL expression was detectable on the surface CRC cells as previously [13], and functional blocking experiments with inhibitory anti-TRAIL RIK2 mAb showed no effect on apoptosis, yet knockdown of TRAIL curtailed CD40-killing. These interesting observations strongly suggest that CD40 signalling induces intracellular TRAIL and subsequent signalling from internal cellular sub-compartments. Although determination of the precise intracellular localisation of TRAIL was beyond the scope of this study, our findings are supported by the reported ability of TRAIL-Rs to undergo TRAIL-mediated endocytosis [43] and signal apoptosis internally via the Golgi apparatus [44]. Our results are also in agreement with reports that DR5 (but not DR4) is critical in TRAIL-mediated apoptosis in CRC cells [45], and in concordance with the overall principle that death TNF ligands can function intracellularly, as exemplified (a) by the ability of FasL to signal Fas-mediated apoptosis via the Golgi and/or ER apparatus [46] and (b) by our previous intriguing observations on the ability of intracellular TRAIL to cause extensive toxicity in cells that are normally totally refractory to extracellular TRAIL (discussed in [33]). Moreover, the activation of TRAIL-mediated apoptosis (and subsequent Bid cleavage) appears to engage a pathway that requires caspase-10 (and not caspase-8) in concordance with the reported ability of caspase-10 to cleave Bid [47] and the functional involvement of caspase-10 [48] and its specific isoforms [49] in TRAIL-mediated apoptosis.

In addition to the critical role for TRAF3 in apoptosis, our results demonstrate an equally central role for the stress kinases p38 and JNK in cell death. Activation of p38/JNK was dependent on intact TRAF3, thus confirming the importance of TRAF3 in a TRAF3/NOX signalling axis that triggers activation of ASK1 and subsequently MKK4 and p38/JNK phosphorylation. p38 and JNK were essential both for direct mitochondrial apoptosis induction and in the simultaneously operating TRAIL/caspase-10/Bid pathway. Yet, their involvement appears to follow defined functional hierarchy, as p38 activation is required for JNK phosphorylation. Although such temporally controlled interplay for p38 and JNK has been previously reported in RCC cells where p38 was dependent on JNK [12], in CRC cells it is p38 that precedes and drives JNK activation, thus providing further evidence for the cell-type specific nature of pro-apoptotic CD40 signalling. 

**Figure 8:**
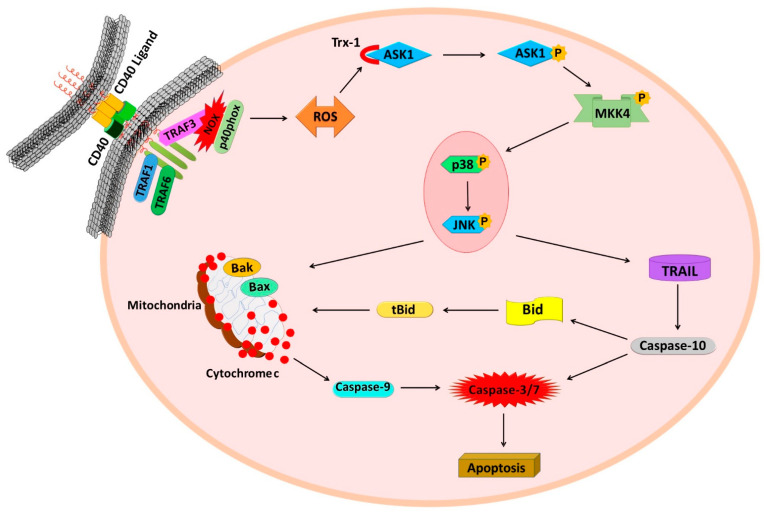
Mechanisms of crosstalk of intrinsic and extrinsic apoptotic signalling pathways in CD40-mediated cancer cell death. Schematic diagram of the mechanisms underpinning mCD40L-mediated colorectal cancer cell apoptosis via crosstalk of intrinsic and TRAIL-associated extrinsic apoptotic signalling. CD40 ligation triggers induction of TRAF3 and activation of NADPH oxidase (NOX) with phosphorylation of the catalytic subunit p40phox. Generation of ROS mediates inactivation of Trx-1 and permits ASK1 activation, subsequent MKK4 phosphorylation, thus leading to phosphorylation of p38 which functionally precedes JNK phosphorylation. This signalling axis then diverges into two pro-apoptotic pathways. The first pathway utilises p38/JNK-mediated induction of ROS-mediated, cyto c/caspase-9-associated, Bak/Bax-engaging mitochondrial apoptosis (the pathway previously detected in other carcinoma cell types). The second pathway triggered by p38/JNK signalling involves rapid induction of intracellular TRAIL (and receptor DR5—not depicted), which causes caspase-10 (but not caspase-8)-dependent apoptosis and is followed by rapid Bid cleavage (tBid induction) thus mediating additional links with mitochondrial apoptosis. Functional experiments suggest that CD40-mediated death is partially dependent on the TRAIL/caspase-10/tBid pathway, thus this pathway crosstalk appears to amplify and accelerate apoptosis in colorectal carcinoma cells in comparison to other tumour cell types (e.g., bladder and renal cancer cells) where no such crosstalk with extrinsic (death receptor) apoptosis has been observed.

In conclusion, our study provides novel mechanistic insights for the enhanced cell death triggered by the mCD40L in CRC cells, as in these cells CD40 signalling entrains two concurrently operating signalling axes. The initial CD40 signal engages TRAF3/NOX/ASK1-signalling and induction of ROS to activate p38 and subsequently JNK. At that point, the p38/JNK signalling ‘hub’ can directly activate the mitochondrial caspase-9-dependent pathway, as also reported in other types of carcinoma cells. However, in CRC cells, p38/JNK also drive rapid TRAIL upregulation to initiate the extrinsic pathway that is triggered by intracellular TRAIL and involves caspase-10-asscociated tBid-activated mitochondrial signalling. Notably, mCD40L-signalling crosstalk with TRAIL-mediated death accounts for approximately half of the extent of apoptosis observed, as evident by the ~50% reduction in apoptosis following (a) pharmacological caspase-10 blockade and more importantly (b) by RNAi-mediated TRAIL and Bid knockdown. By engaging both intrinsic and extrinsic apoptotic signalling to rapidly activate mitochondrial via two signals simultaneously, CD40 can amplify and accelerate apoptotic signalling (Figure 8). Thus, our findings have further unravelled the multifaceted pro-apoptotic properties and unique nature of the CD40/mCD40L dyad, whilst also providing novel evidence of the direct TNFR crosstalk that drives rapid carcinoma cell death, which has implications for the use of CD40 as an increasingly attractive therapeutic target in cancer [50].

## Data Availability

Not applicable.

## Supplementary Materials

The following supporting information can be downloaded at: https://www.mdpi.com/article/10.3390/cells11203274/s1, Figure S1: TRAF3 knockdown by stable shRNA expression and its effect on mCD40L-mediated apoptosis; Figure S2: Down-regulation of Trx-1 expression by mCD40L; Figure S3: The role of Nox activity in mCD40L-mediated pro-apoptotic signalling; Figure S4: Bax knockdown by stable shRNA expression and its effect on mCD40L-mediated apoptosis.
