# Supplementary figures and images for: TRAF3/p38-JNK Signalling Crosstalk with Intracellular-TRAIL/Caspase-10-Induced Apoptosis Accelerates ROS-Driven Cancer Cell-Specific Death by CD40
Source: Cells. 2022 Oct 18;11(20):3274. doi: 10.3390/cells11203274 (PMC9600997; doi:10.3390/cells11203274)

Figure S1

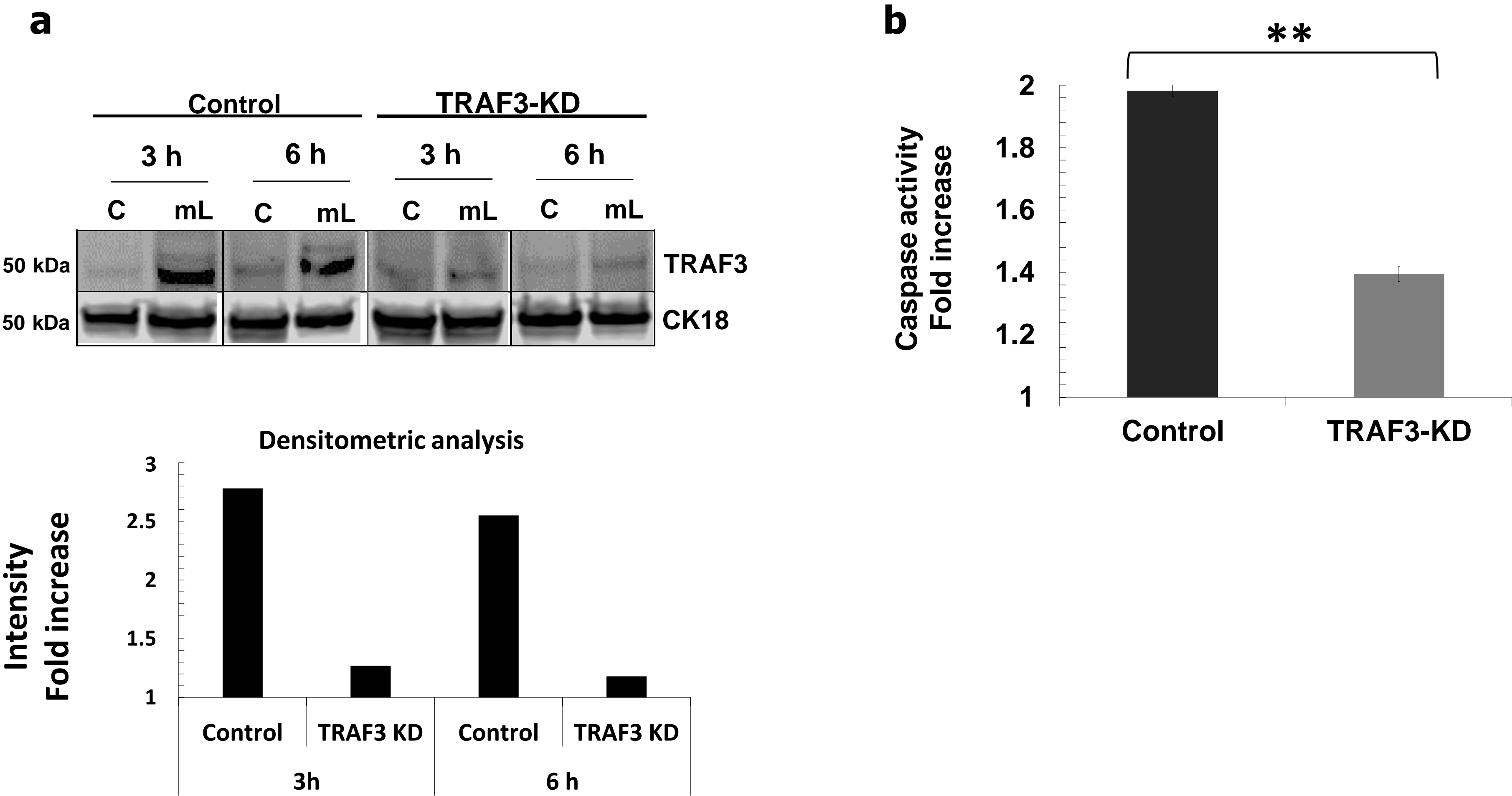

Figure S2

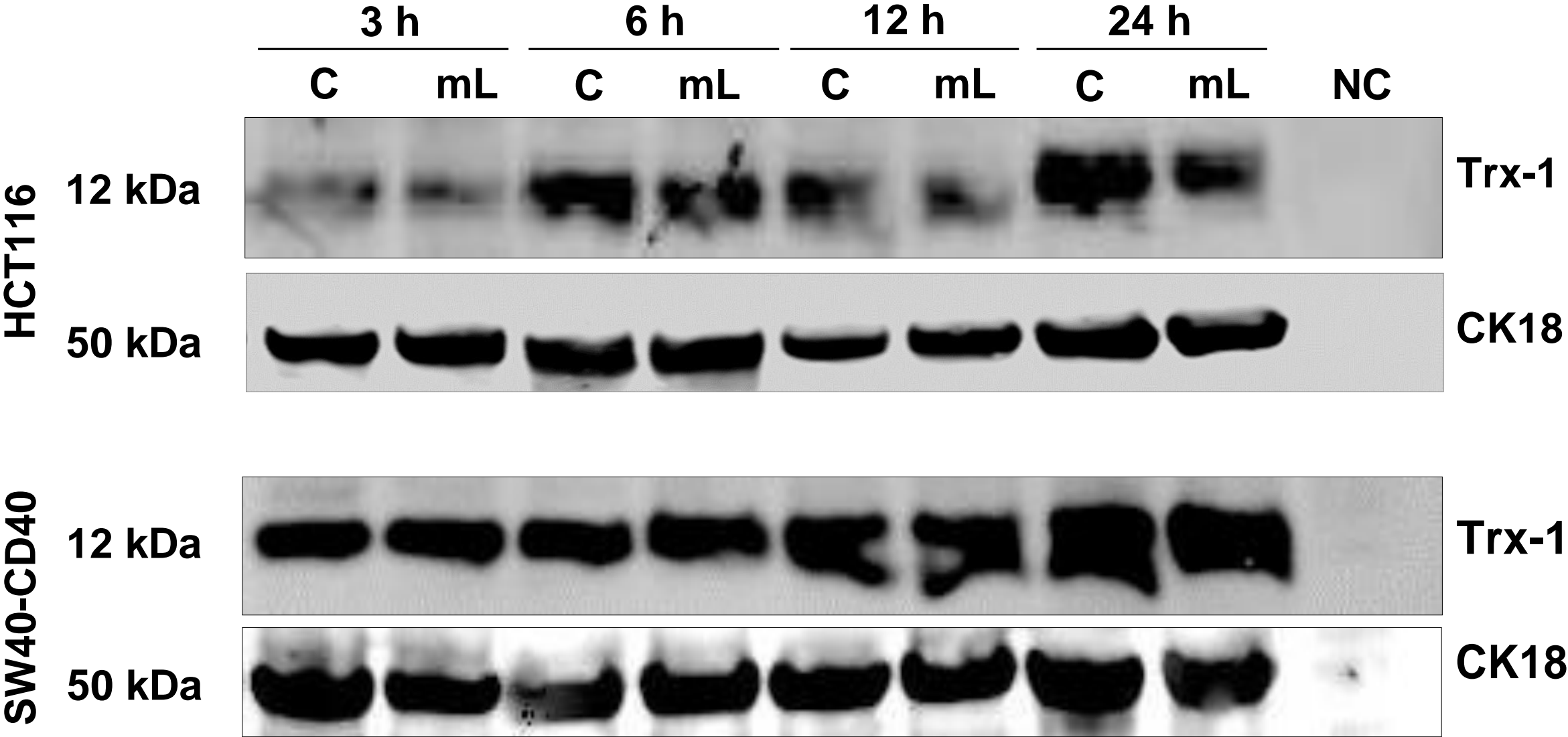

Figure S3

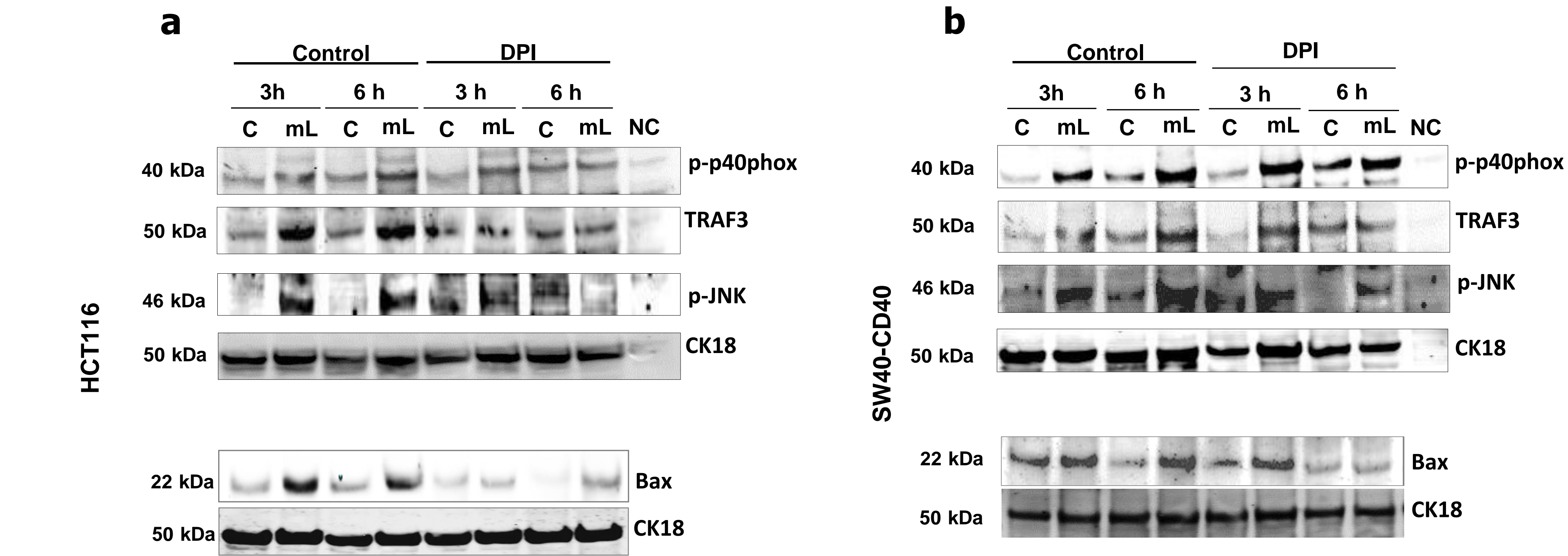

Figure S4

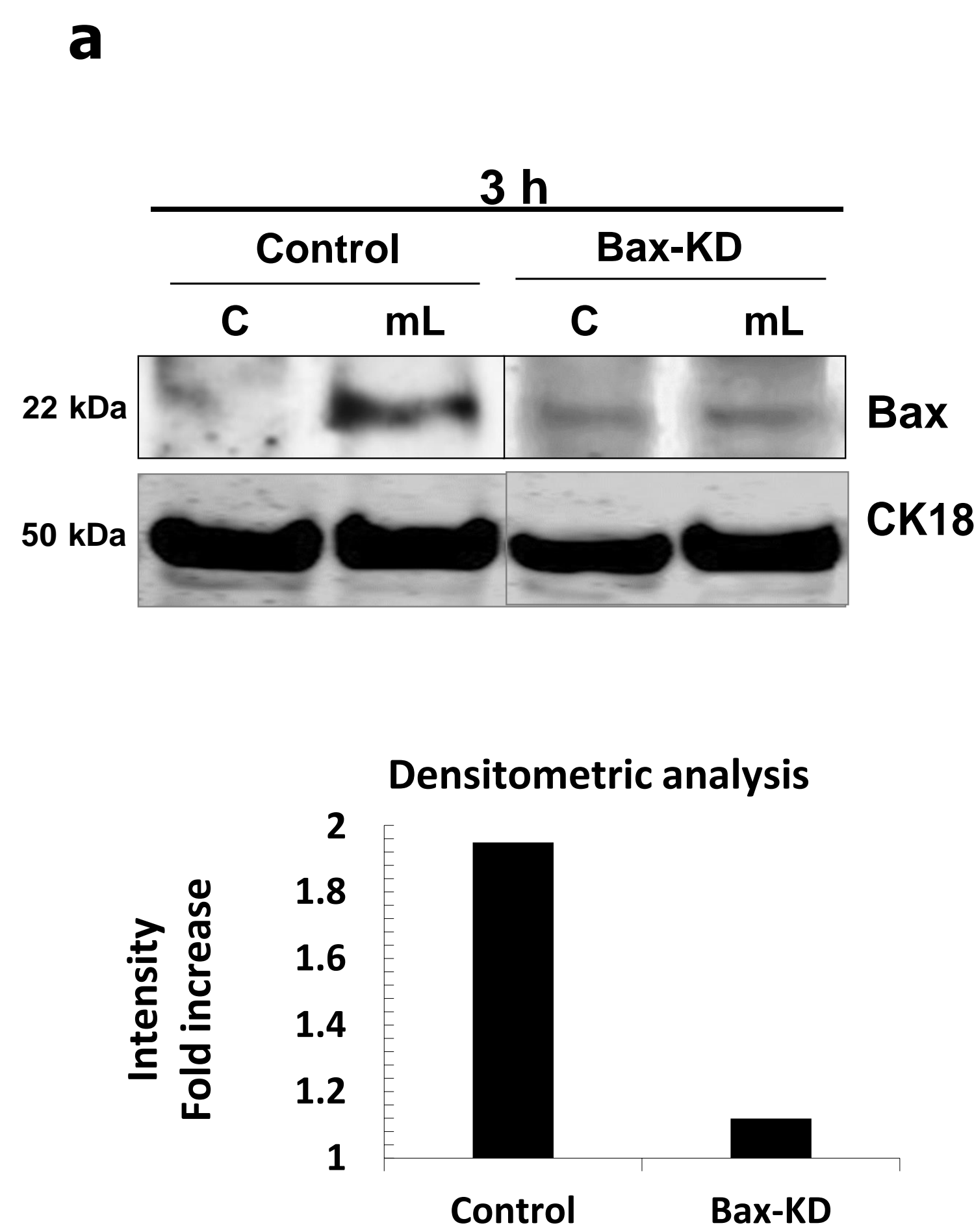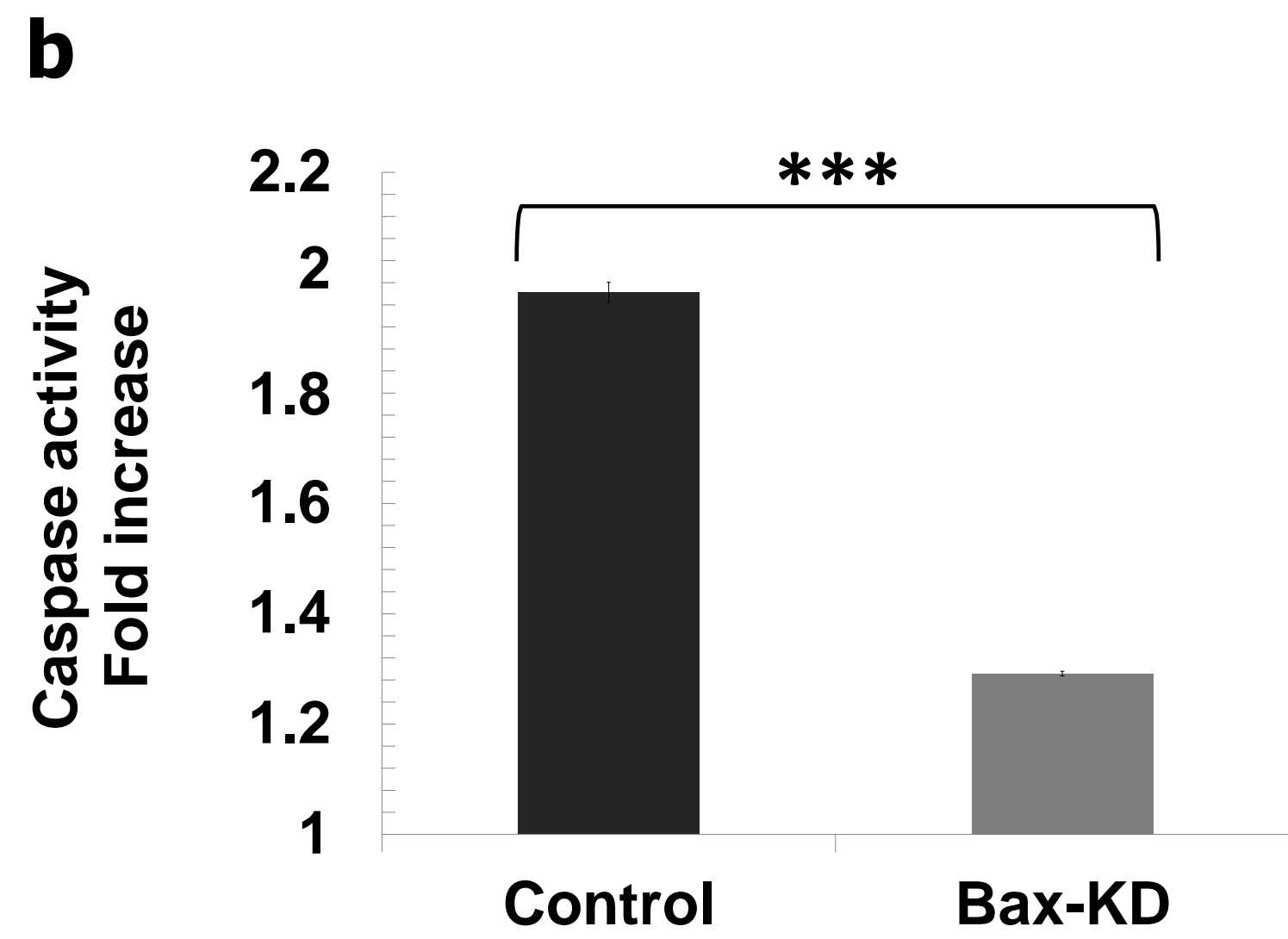

Supplement: Supplementary file 1 [file cells-11-03274-s001.zip › cells-1956389-supplementary.pdf]
